# In Memory of Professor Jung Hyun Seul, MD, PhD (1942–2025): Pioneer, Teacher, Mentor, and Gentle Giant of Korean Plastic Surgery

**DOI:** 10.1055/a-2688-3936

**Published:** 2025-09-18

**Authors:** Jun-Ho Lee, Yong-Ha Kim

**Affiliations:** 1 Department of Plastic and Reconstructive Surgery, Yeungnam University College of Medicine, Daegu, South Korea

On March 26, 2025, Professor Jung Hyun Seul peacefully passed away, leaving behind a legacy that has profoundly shaped Korean plastic surgery. He was not merely a skilled surgeon or esteemed educator—he was a visionary leader, a devoted mentor, and a man of deep human warmth whose influence resonates across generations.

## A Founding Figure and Academic Pioneer

Born in Daegu in 1942, Professor Seul graduated from Korea University College of Medicine in 1967 and completed his surgical and plastic surgery training at Severance Hospital, Yonsei University. In 1976, he became Korea's 29th board-certified plastic surgeon and later earned his PhD in Medicine in 1980, solidifying his academic foundation.

In 1983, as Yeungnam University College of Medicine was established, he became the founding chair and head of the Department of Plastic and Reconstructive Surgery. For over 23 years until his retirement in 2006, Professor Seul led the department with unwavering dedication, laying the groundwork for what has become one of Korea's leading centers of plastic surgery education and clinical care.

## A Trailblazer in Breast Reconstruction

While Professor Seul contributed across a wide range of surgical fields, his groundbreaking work in breast reconstruction was especially transformative. In 2000, he became the first surgeon in Korea to complete over 100 cases of breast reconstruction—a remarkable achievement that demonstrated both his technical expertise and his commitment to restoring not just form, but dignity and wholeness to his patients.

His 2005 textbook, *Plastic Surgery of the Breast* , was the first of its kind, authored by a Korean surgeon for Korean patients and professionals. At a time when most academic resources were only available in English, this pioneering work bridged cultural and linguistic gaps, localizing global surgical knowledge. Recognized for its academic excellence and clinical relevance, the book earned him the prestigious 38th Dong-A Medical Literature Award.

Thanks to his teaching and leadership, many of his students have gone on to specialize in breast reconstruction, and Yeungnam University Medical Center continues to be recognized nationally for excellence in this field.

## Leadership, Service, and Global Recognition

Beyond the operating room and lecture hall, Professor Seul played a critical role in advancing plastic surgery as a profession. He served as the President of the Korean Society of Plastic and Reconstructive Surgeons (1996-1997) and the Korean Society of Aesthetic Plastic Surgery (1998-1999). In May 1999, as the President of the Oriental Society of Aesthetic Plastic Surgery (OSAPS), he successfully hosted international conferences in Seoul and contributed to expanding Korea's presence in the global medical community.

From 2001 to 2023, he also served as the 10th Director of Yeungnam University Medical Center, demonstrating outstanding leadership in health care administration. His impact extended from surgical excellence to institutional development.

## A Man of Character and Heart

What truly set Professor Seul apart was his humanity. Though disciplined and principled, he was also generous, humorous, and full of life. His students remember not only his academic rigor but also his warmth—his readiness to share a drink, sing a song at karaoke, or offer a thoughtful word of encouragement. With his big hands, dignified presence, and compassionate heart, he embodied both strength and grace.

Even during his final illness, he retained his dignity, showing deep consideration for those around him. To the very end, he was a teacher—not only of surgery but of how to live with honor and purpose. He also donated development funds to Yeungnam University Medical Center and the Korean Society of Plastic and Reconstructive Surgeons, where he served.

He raised not only surgeons, but thinkers and leaders. Since he founded the department in 1983, the Department of Plastic Surgery at Yeungnam University Hospital has trained 61 Plastic Surgery Boardmen, 22 PhDs, and 43 master' graguates. His legacy lies not only in his skills and titles, but also in the lives he touched and transformed.

## A Lasting Legacy

Professor Seul will be remembered not only as a pioneer in the field of plastic surgery, but as a rare kind of human being—principled yet kind, disciplined yet warm. To his students, he was not only a professor but a father figure and a guiding star.

His contributions will continue to echo through the halls of Yeungnam University, through the hands of his students, and through every patient whose life has been healed and uplifted.

We honor him. We mourn him. And we carry forward his spirit, with gratitude and reverence.

May he rest in eternal peace.

Seul Jung Hyun

